# Sequencing and Analysis of mtDNA Genomes from the Teeth of Early Medieval Horses in Poland

**DOI:** 10.3390/genes17010095

**Published:** 2026-01-18

**Authors:** Edyta Pasicka, Mateusz Baca, Danijela Popović, Daniel Makowiecki, Maciej Janeczek

**Affiliations:** 1 Department of Biostructure and Animal Physiology, Wrocław University of Environmental and Life Sciences, Kożuchowska 1, 51-631 Wrocław, Poland; maciej.janeczek@upwr.edu.pl; 2 Centre of New Technologies, University of Warsaw, S. Banacha 2c, 02-097 Warsaw, Poland; m.baca@cent.uw.edu.pl (M.B.); dpopovic@cent.uw.edu.pl (D.P.); 3 Institute of Archaeology, Nicolaus Copernicus University in Toruń, Szosa Bydgoska 44/48, 87-100 Toruń, Poland; makdan@umk.pl

**Keywords:** zooarchaeology, equine dentition, aDNA, archaeological sites, Poland

## Abstract

Background: This study presents the sequencing and analysis of mitochondrial DNA (mtDNA) genomes from nine early medieval horse remains excavated across archaeological sites in Silesia region in present day Poland. Methods: Using aDNA extraction protocols optimized for short fragments, combined with target enrichment and high-throughput sequencing, we reconstructed partial mtDNA sequences for seven of the specimens. Results: The authenticity of the aDNA was confirmed through damage pattern analysis. Phylogenetic reconstruction revealed that the specimens belonged to six distinct mtDNA lineages (B, D, E, G, L, and M), indicating a high level of mitochondrial diversity within medieval Silesian horse population. Conclusions: These findings highlight the extensive mtDNA variability among domestic horses, reflecting the diversity of their ancestral populations rather than modern breed differentiation. This research enhances our understanding of horse population structure in medieval Europe, emphasizing the genetic complexity present during this period.

## 1. Introduction

Archaeozoological research investigates the interactions between humans and animals in the past. Traditional methods in this field enable the identification of animal species found at archaeological sites, as well as determinations of their sex and lifestyle [1,2,3,4]. Examining marks on bones provides valuable insights into practices such as meat processing and reveals how animals were treated—whether as objects of worship or victims of predators [1,2,3,4,5,6,7]. Isotopic analysis of bone chemistry allows researchers to track animal movements during their lifetimes, shedding light on seasonal migrations and dietary habits [8]. Recent advances in ancient DNA research have significantly enhanced our ability to extract and analyze genetic data from human and animal bones and teeth, leading to a deeper understanding of their relationships throughout history [9,10,11]. Additionally, new molecular genetic technologies serve as important supplements to excavation studies, helping to determine the timing and location of horse domestication [9,10,11,12]. The domestication of wild horses in the early 4th millennium BCE on the southeastern European steppes marked a pivotal development—transforming warfare, facilitating the use of horse-drawn vehicles, and advancing wheeled transportation [13,14].

Regarding the Western Slavs, including the tribes of early medieval Poland (10–13th c.), historical, archaeozoological, and archaeological sources indicated the significant importance of the horse in military, religious, and social life [5,15]. Even earlier evidence of Slavic horse use includes Procopius (6th century), who noted Slavic horsemen among Danube tribes [16,17], and Theophylact Simocatta (early 7th century), who described elite Slavs riding horses [18]. Early 10th-century customs regulations from Austria taxed Slavs from Bohemia and Rus on horse trade [19]. The Jewish traveler Ibrahim ibn Yaqub (~966 AD) reported abundant horses and active trade among the Slavs of Nakon [19], indicating they bred high-quality horses valued for both performance and appearance. Mieszko I, Poland’s founder, equipped warriors with horses, emphasizing horsemanship as a key aspect of warfare [19,20,21]. Horses also provided meat, mare’s milk (kumys), and hides; bones were repurposed for skates and sled runners [22,23]. Given the high cost of horse ownership, owning horses signified social elite status. Both historical accounts and zooarchaeological findings highlight the horse’s religious and cultural importance among the Slavs. This article presents the analysis of mtDNA genomes of early medieval horses excavated from various archaeological sites in Silesia region, along with efforts to interpret the results.

The above outline of knowledge about early medieval horses has recently been expanded through paleogenomic analyses of 60 horses from various regions [24,25]. These studies indicate that, between the 10th and 13th centuries, horses belonging to 16 different haplotypes were used in Poland. Among the most common haplotypes were L, B, and M, which accounted for 25%, 17%, and 13% of the samples, respectively. Although three horses from Silesia were included in the sample set, no mitochondrial DNA data was obtained from them. Therefore, this article aims to address this gap by presenting the results of research conducted on nine samples from Silesia. This region was historically one of the most politically, socially, and economically significant areas of the Western Slavs. Culturally, Silesia served as a bridge between southern Europe and northern regions, including the Baltic zone and the areas associated with the origins of the earliest Polish state.

## 2. Materials and Methods

### 2.1. Characteristics of the Sample Collection

**Figure 1:**
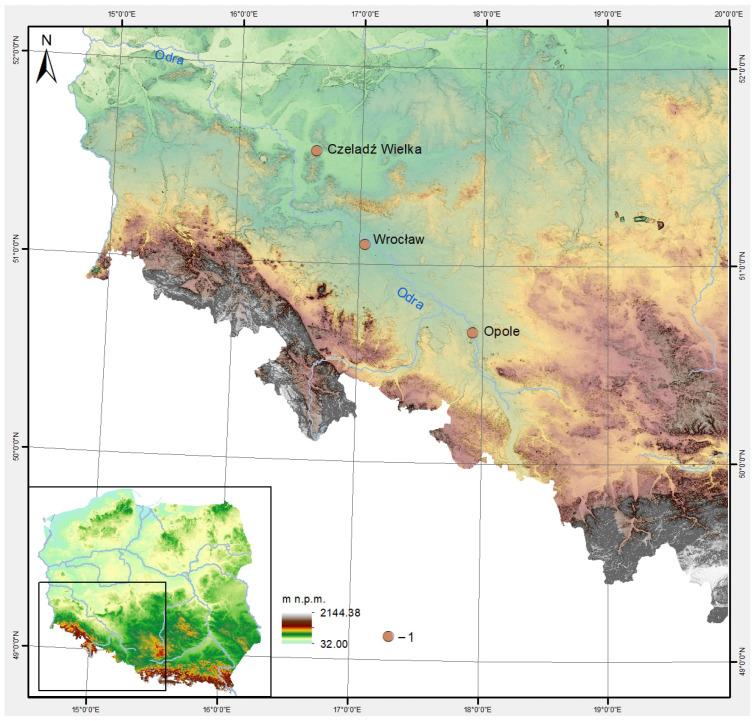
The research materials collected from early medieval archaeological sites in Poland (drawing by M. Skrzatek).

Sequencing and analysis of mtDNA genomes were undertaken for teeth of nine early medieval horses belonging to the collection of the Archaeozoology Laboratory and Museum of Standards, Department of Biostructure and Animal Physiology at the Wrocław University of Environmental and Life Sciences in Poland. These selected samples were from the early medieval horses (10–13th c.) from the main archaeological sites in Silesia, Poland. Seven samples were collected from Wrocław, one from Opole, and one from Czeladź Wielka (Table 1, Figure 1; Supplementary Materials; Table S1). Horse samples were dated based on the archaeological context and chronostratigraphy of the layers in which they were found, as well as the archaeological materials recorded within them, primarily pottery and other artefacts of material culture [26,27]. As a routine, the AMS 14C method was also used to verify archaeological dating. Two samples were dated using this method at the Poznan Radicarbon Laboratory. The dates were calibrated with OxCal ver. 4.4.4 software [28], using the IntCal20 radiocarbon calibration curve [29]. Calibration of BP dates confirmed the archaeological chronology of both samples (cf. Supplementary Materials; Table S1).

### 2.2. mtDNA Genomes Sequencing and Analysis

All experimental procedures were performed in a laboratory dedicated to ancient DNA work at the Laboratory of Paleogenetics and Conservation Genetics, Centre of New Technologies, University of Warsaw. Strict precautions were taken to avoid contamination. This work was conducted in a dedicated pre-PCR laboratory, where no modern, amplified, or cloned DNA was ever introduced. All personnel wore disposable lab coats, face shields, and gloves. Working areas were regularly treated with 6% bleach and DNA ExitusPlus (Applichem, Darmstadt, Germany) and were UV-irradiated when not in use. All reactions were carried out in PCR cabinets equipped with HEPA filters. Only filter tips and sterile disposables were used. A negative control containing no bone powder was processed alongside each batch of samples to monitor potential contamination. Prior to extraction, the teeth were first thoroughly rinsed with 0.5% NaOCl (bleach), then with water, and subsequently UV-irradiated from all sides. Approximately 50 mg of tooth powder was drilled from the surface of the tooth using Dremel tool. DNA was extracted from the tooth powder according to a protocol optimized for retrieval of short DNA molecules [30]. DNA extracts were converted into double-stranded, double-indexed sequencing libraries according to the protocol of Meyer and Kircher [31], with minor modification: after blunt-end repair and fill-in, enzymes were heat-inactivated instead of using SPRI purification. For each sample, two independent sequencing libraries were prepared. Indexing PCR were run in triplicate for each sequencing library. After amplification, PCR products were pooled and SPRI purified. In the ancient DNA extracts, the amount of endogenous DNA is minute, very often under 1%, which makes direct sequencing of DNA extract implausible. To obtain mtDNA sequence we used target enrichment procedure which allows to increase the amount of certain DNA fragment in DNA extract. As a bait in enrichment reactions, we used mtDNA of modern domestic horse (*Equus caballus*) enzymatically modified according to protocol proposed by Horn [32]. Following the same protocol, we performed two rounds of overnight hybridization. After each hybridization post-capture PCR was performed to increase the amount of DNA. Enriched sequencing libraries were quantified, pooled in equimolar ratio, and sequenced on NextSeq550 Illumina platform (MidOutput kit, 2 × 75 cycles, Illumina, San Diego, CA, USA). Raw reads were demultiplexed based on index sequences using bcl2fastq Conversion Software v2.20 (Illumina, San Diego, CA, USA). Adapter sequences were trimmed and paired-end reads were collapsed using AdapterRemoval v2 [33]. Merged reads were mapped to the reference mitochondrial genome of domestic horse (GenBank NC_001640) using mem algorithm from bwa 0.7.17 [34]. Only reads with mapping quality over 30 and longer than 30 bp were retained. Duplicates were removed; variants and consensus mtDNA sequences were called using Samtools and Bcftools [35]. We called only positions with a minimum 3× coverage. Each bam alignment was inspected manually in Tablet [36].

We subsequently mapped sequencing reads to the reference domestic horse nuclear genome (EquCab2) and used Zonkey pipeline [10] to determine sex of studied specimens. Endogenous ancient DNA molecules typically exhibit excess of deaminated cytosine towards the ends of molecules, so we checked for this pattern using MapDamage v.2 [37].

To reveal the phylogenetic position of analyzed horse remains we reconstructed Maximum Likelihood phylogeny using a set of 150 mtDNA genomes of contemporary domestic horses [9,38]. The alignment has length of 16,439 bp. Phylogeny was reconstructed in RAxML [39] using GTRGAMMA substitution model. To assess branch support, we used rapid bootstrap analysis with an automatic bootstrap convergence test using Majority Rule Extended (-# autoMRE). The bootstrap converged after 350 replicates. To check the haplotype sharing pattern we used DNAsp v. 6.12 [40].

## 3. Results and Discussion

We reconstructed partial mtDNA sequence in the case of seven specimens with mean coverage ranging from 9 to 69× (Table 2). These data allowed the reconstruction of large portions of the mitogenome sequences (0.85–0.99 of the reference sequence), providing sufficient information for robust phylogenetic inference [41]. In the case of two specimens the number of reads mapping to the horse mtDNA was too low to reconstruct even partial mtDNA genome. The first step of the analysis was the verification of authenticity of obtained aDNA sequences. In each case the fraction of deaminated nucleotides at 3′ and 5′ ends of DNA molecules was higher than 10% and the mean length of mtDNA fragments was below 100 bp suggesting that we obtained genuine ancient DNA sequences (Table 2). Mapping to the EquCab2 horse nuclear reference genome yielded between 266 and 489,000 unique reads. In case of eight specimens this was more than 5000 reads what allowed for reliable sex determination using the Zonkey pipeline [10] in case of seven specimens (Table 2). For the EQW010 specimen, the Zonkey pipeline yielded an inconclusive result (Supplementary Figure S1). It yielded 0.0013× mean genome coverage across autosomes and 0.0012× mean coverage of X chromosome suggesting it was a female. However, the sex of this specimen could not be determined morphologically, so we considered the sex of this sample unknown. In most archaeological assemblages the sex ratio of horse remains is highly biased towards males [24,42], reflecting the way horses were utilized in medieval societies. The male ratio here was 0.715 and was not different from the male ratio reported for other Polish medieval populations [24] (Fisher’s exact test; *p* = 0.67).

**Figure 2:**
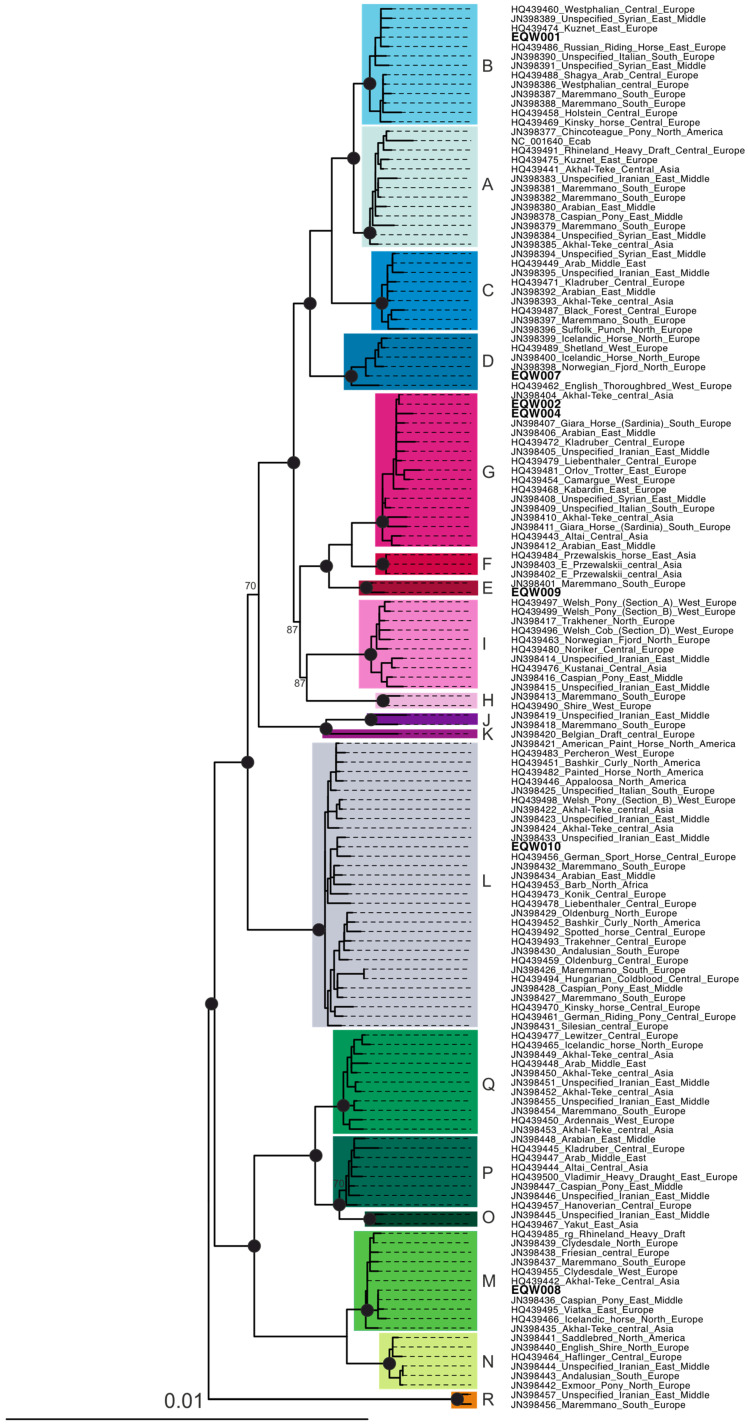
Maximum Likelihood phylogeny of domestic horses based on 150 mtDNA genomes. The tree was reconstructed in RaxML and rooted with *Equus asinus* mtDNA genome (Table S2, Figure S1). Black circles denote bootstrap support for the node higher than 90, otherwise the bootstrap value is indicated. Specimens analyzed in this study are bolded. Tips are annotated with GenBank accession, breed name and region of origin.

The reconstructed mtDNA phylogeny revealed 18 well-supported mtDNA lineages in line with previous findings (Figure 2) [9,38]. The phylogenetic position of the studied samples allowed for their unambiguous classification to the known mtDNA lineages (Table 2). To confirm the classification, we used MitoToolPy-seq script [43] which allowed us to assign mtDNA sequence to a specific lineage; in all cases, classification based on the phylogenetic position was concordant with result from MitoToolPy-seq. Each of the seven specimens that yielded an mtDNA sequence belonged to a distinct mtDNA sublineage, and altogether six main lineages (B, D, E, G, L and M) were represented. This suggests a high mtDNA diversity among domestic horses from the relatively limited geographic area, although similar diversity was previously found on other medieval sites in Poland, e.g., seven haplogroups were recorded among ten specimens from Kruszwica site and five haplogroups among five individuals from Ostrów Lednicki [24]. Although the mtDNA diversity of horses is highest amongst the domesticated animals, it reflects the high diversity of the ancestral *Equus ferrus* that underwent domestication rather than partitioning associated with modern breed formation. Researchers concur that the considerable diversity of mtDNA haplotypes found in fossil remains stems from the domestication of numerous mares possessing diverse haplotypes [44,45,46]. Asia exhibits the highest variability in mitochondrial genome sequences, with the prevalent haplogroups being G, Q, and A. In contrast, these haplogroups are less common in the Middle East and Europe, where haplogroup L is predominant [9,47]. In Popović et al. [24] Polish medieval horses were grouped together with other ancient and modern domestic horses, showing no significant differentiation between different populations and regions.

**Figure 3:**
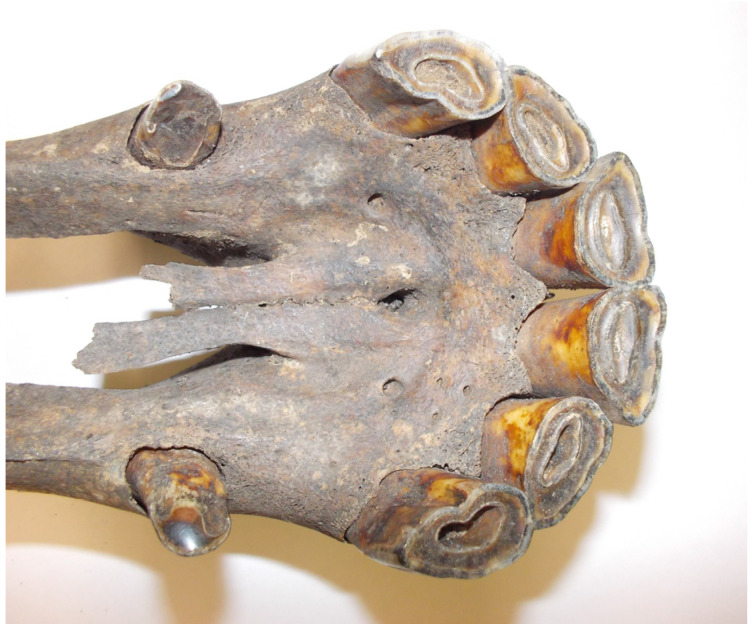
Early medieval horse (sample EQW002) from Wrocław, Nowy Targ (Photo by E. Pasicka).

Compared to available sequences of modern horses, sample EQW002 (Figure 3) has an identical haplotype to the Central Asian sample (Akhal-Teke breed, sample akT06 in Achilli et al. [9]) and sample EQW008 has an identical haplotype with Middle Eastern Caspian Pony breed (JN398436). An identical haplotype found in two specimens may suggest a recent common ancestor; however, no association between the mtDNA lineage and modern horse breads, nor the geographic provenience of the horse, has been found [9,38]. Therefore, inferences of close relationships based solely on mtDNA haplotype sharing should be made with extreme caution, as identical haplotypes may also arise through homoplasy.

**Figure 4:**
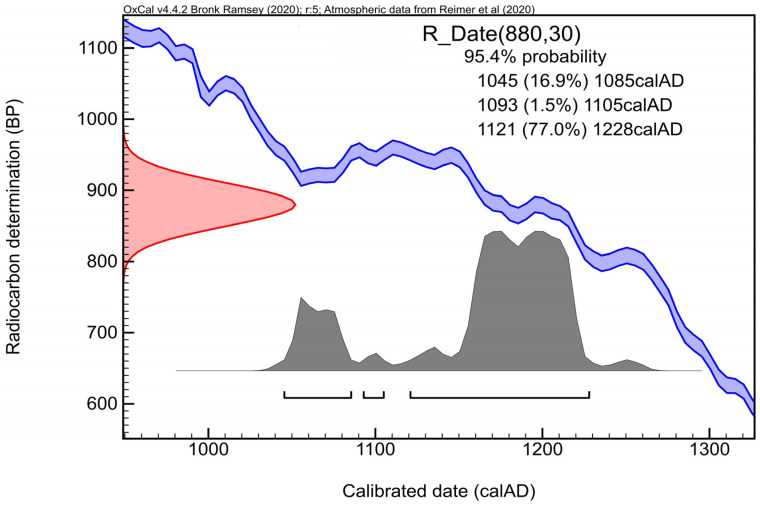
Wrocław-Nowy Targ, AMS 14C chronology of the horse tooth (EQW002). OxCal v4.4.2 Ramsey [51]; Atmospheric data from Reimer et al. [29].

Radiocarbon dating of the tooth sample EQW002 indicated the death of the individual at 880 ± 30 BP (Poz-162263) (Poznań Radiocarbon Laboratory), and thus with a probability of 95.4% in the range 1045–1228 CE (Figure 4). Unfortunately, the find should not be linked with the Mongol invasion of Poland in 1241 CE [48,49]. Therefore, we presume on another explanation. At that time, Nowy Targ, where the samples were found, was part of a rapidly developing stronghold centre located on Ostrów Tumski [27]. Long-distance trade routes from east to west and south to north crossed here from at least the 10th century [50]. It is therefore possible that the horses we discovered of eastern Asian and Caspian origin are the result of trade contacts.

Thus, our research complements existing mtDNA data on horses from the Silesian region in Poland, expanding our understanding of horse population structure in medieval Europe and highlighting the considerable genetic diversity and complexity characteristic of this period.

## 4. Conclusions

Our findings highlight the extensive mtDNA variability among domestic horses from medieval sites in Silesia, although it is not significantly higher than that observed in other contemporary populations from the region. This pattern likely reflects the genetic diversity of their ancestral populations rather than modern breeds differentiation. Overall, this study contributes to a better understanding of horse population structure in medieval Europe, emphasizing the considerable genetic complexity present during that period.

## Figures and Tables

**Table 1:** Details of samples used for genetic analyses.

No	Lab ID	Site	Social Context	Chronology	Skeletal Element
1	EQW001	Opole-Ostrówek	stronghold	10–12th c.	Skull
2	EQW002	Wrocław-Nowy Targ	settlement	11–early 13th c.	Skull
3	EQW003	Czeladź Wielka	settlement	9/10–13th c.	Skull
4	EQW004	Wrocław-Ostrów Tumski	stronghold	12/13th c.	Mandible
5	EQW006	Wrocław-Ostrów Tumski	stronghold	10–13th c.	Skull
6	EQW007	Wrocław-Ostrów Tumski	stronghold	12–13th c.	Skull
7	EQW008	Wrocław-Ostrów Tumski	stronghold	Early Medieval?	Mandible
8	EQW009	Wrocław-Nowy Targ	settlement-town	11–15th c. (18th c.)	Mandible
9	EQW010	Wrocław-Nowy Targ	settlement-town	11–15th c. (18th c.)	Mandible

**Table 2:** Details of mtDNA enrichment, sequencing, and sex determination.

No	Lab ID	# Reads	mtDNA Unique Reads	Mean mtDNA Coverage	mtDNA Bases Covered	mtDNA Hg	5′/3′ DNA Damage	EquCab2 Unique Reads	Genetic Sex
1	EQW001	466,294	6795	35.13	16,557	B1c	0.16/0.17	15,796	male
2	EQW002	1,351,970	11,702	69.68	16,550	G1a_JN398404	0.17/0.16	489,608	male
3	EQW003	2,153,524	91	n.a.	n.a.	n.a.	n.a.	266	n.a.
4	EQW004	2,480,848	3346	17.98	16,284	G1a	0.09/0.11	225,032	male
5	EQW006	1,134,316	69	n.a.	n.a.	n.a.	n.a.	6982	female
6	EQW007	2,401,196	3625	20.68	16,026	D1a2	0.15/0.16	41,255	male
7	EQW008	4,045,447	3920	20.68	16,381	M1b	0.19/0.15	256,250	male
8	EQW009	2,164,675	1779	14.86	14,871	E	0.16/0.17	211,223	female
9	EQW010	3,199,186	1821	9.93	14,167	L3a1	0.17/0.16	37,528	inc.

# reads—number of raw sequencing reads, mtDNA unique reads—number of unique reads mapping to the mtDNA reference, mean mtDNA coverage—mean coverage of mtDNA genome, 5′/3′ DNA damage—fraction of deaminated nucleotides at 5′ and 3′ end of DNA molecules, EquCab2 unique reads—number of unique reads mapping to the EquCab2 reference genome. n.a.—not applicable, inc.—inconclusive.

## Data Availability

The raw data used in this study has been deposited in RepOD repository (doi:10.18150/SDVQQV).

## Supplementary Materials

The following supporting information can be downloaded at: https://www.mdpi.com/article/10.3390/genes17010095/s1, Table S1: List of sites with early medieval horse remains [25,26,27,52], Table S2: List of modern genomes with appropriate metadata [9,38], Figure S1_Zonkey_output_for_EQW010-1 [35,53,54,55,56].
