# Supplementary material for: Sequencing and Analysis of mtDNA Genomes from the Teeth of Early Medieval Horses in Poland
Source: Genes (Basel). 2026 Jan 18;17(1):95. doi: 10.3390/genes17010095 (PMC12841091; doi:10.3390/genes17010095)
Supplement: Supplementary file 1 [file genes-17-00095-s001.zip › genes-4000137-supplementary/Figure_S1_Zonkey_output_for_EQW010-1.pdf]

PALEOMIX ZONKEY V1.3.7 - DB REV. 20161101

A PIPELINE FOR DETECTION OF F1 HYBRIDS IN EQUIDS.

Schubert M, Ermini L, Sarkissian CD, Jónsson H, Ginolhac A, Schaefer R, Martin MD, Fernández R, Kircher M, McCue M, Willerslev E, and Orlando L. "**Characterization of ancient and modern genomes by SNP detection and phylogenomic and metagenomic analysis using PALEOMIX**". Nat Protoc. 2014 May;9(5):1056-82. doi:10.1038/nprot.2014.063. Epub 2014 Apr 10. PubMed PMID: 24722405.

CONTENTS

Top
Introduction
Analysis overview
Reference Panel
Admixture Estimates
PCA Plots
Treemix Analyses
References

INTRODUCTION

The Zonkey Pipeline is a easy-to-use pipeline designed for the analyses of low-coverage, ancient DNA derived from historical equid samples, with the purpose of determining the species of the sample, as well as determining possible hybridization between horses, zebras, and asses. This is accomplished by comparing one or more samples aligned against the *Equus caballus* 2.0 reference sequence with a reference panel of modern equids, including wild and domesticated equids.

For more information, please refer to the the documentation for the Zonkey pipeline or the documentation for the PALEOMIX pipeline, on which the Zonkey pipeline is based.

ANALYSIS OVERVIEW

Zonkey run using database rev. 20161101. Data processed using pysam v0.16.0.1, SAMTools v1.9.0 [*Li et al. 2009*] and PLINK v1.90 [*Purcell et al. 2007*]; plotting was carried out using R v3.6.0. Additional tools listed below.

Nuclear report from '*/workspace1/NEXTSEQ_RUNS/NS027_analiza/Equus_WROCLAW/EQW010A1L1_S106_EquCab2_nodup.bam*'

Number of reads processed:	35089
Number of reads overlapping SNPs:	22332
Number of SNPs used (incl. transitions):	22279
Number of SNPs used (excl. transitions):	6642

Autosomes vs. sex-chromosomes:

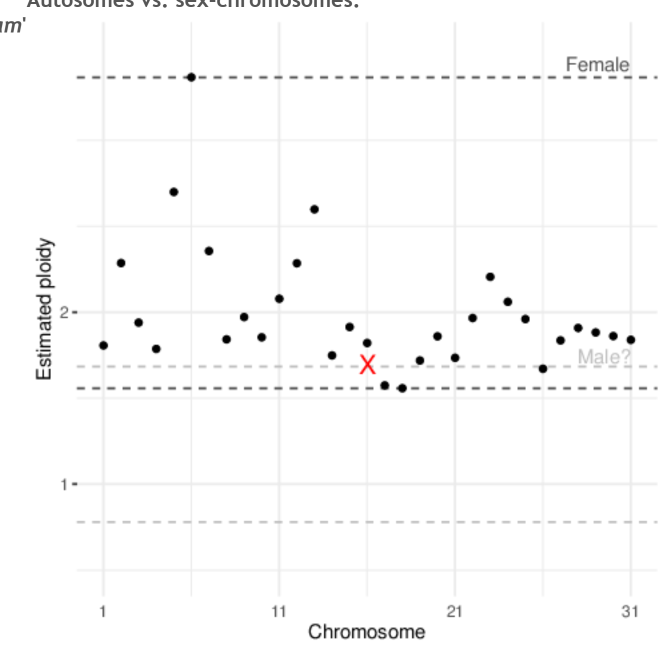

REFERENCE PANEL

ID	Group(2)	Group(3)	Species	Sex	Sample Name	Publication
AAsi	Ass	NonCaballine	*E. a. asinus*	Male	Willy	doi:10.1038/nature12323
AKia	Ass	NonCaballine	*E. kiang*	Female	KIA	doi:10.1073/pnas.1412627111
AOna	Ass	NonCaballine	*E. h. onager*	Male	ONA	doi:10.1073/pnas.1412627111
ASom	Ass	NonCaballine	*E. a. somaliensis*	Female	SOM	doi:10.1073/pnas.1412627111
HCab	Horse	Caballine	*E. caballus*	Male	FM1798	doi:10.1016/j.cub.2015.08.032
HPrz	Horse	Caballine	*E. przewalskii*	Male	SB281	doi:10.1016/j.cub.2015.08.032
ZBoe	Zebra	NonCaballine	*E. q. boehmi*	Female	BOE	doi:10.1073/pnas.1412627111
ZGre	Zebra	NonCaballine	*E. grevyi*	Female	GRE	doi:10.1073/pnas.1412627111
ZHar	Zebra	NonCaballine	*E. z. hartmannae*	Female	HAR	doi:10.1073/pnas.1412627111

ADMIXTURE ESTIMATES

Admixture proportions estimated using ADMIXTURE v1.3 *[Alexander et al. 2009]*, using default parameters.

2 ancestral groups

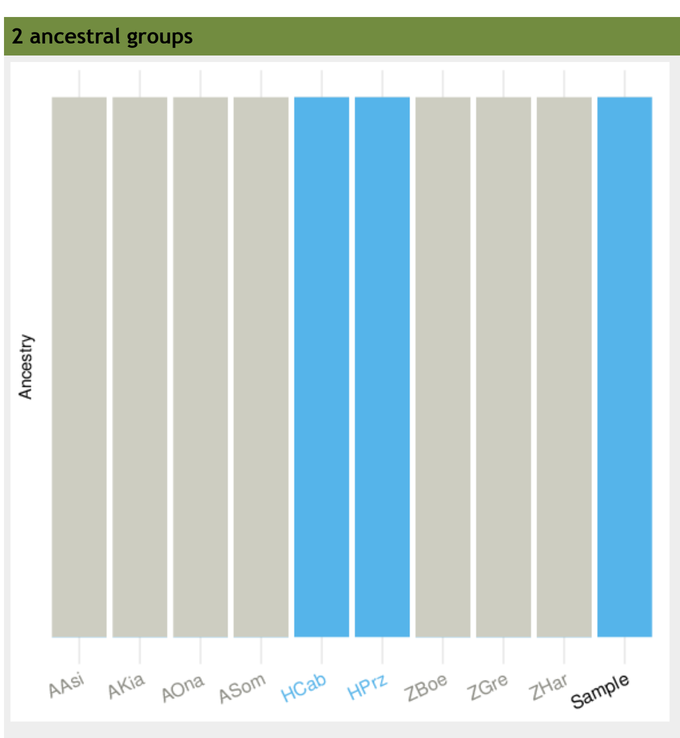

No admixture detected.

2 ancestral groups, excluding transitions

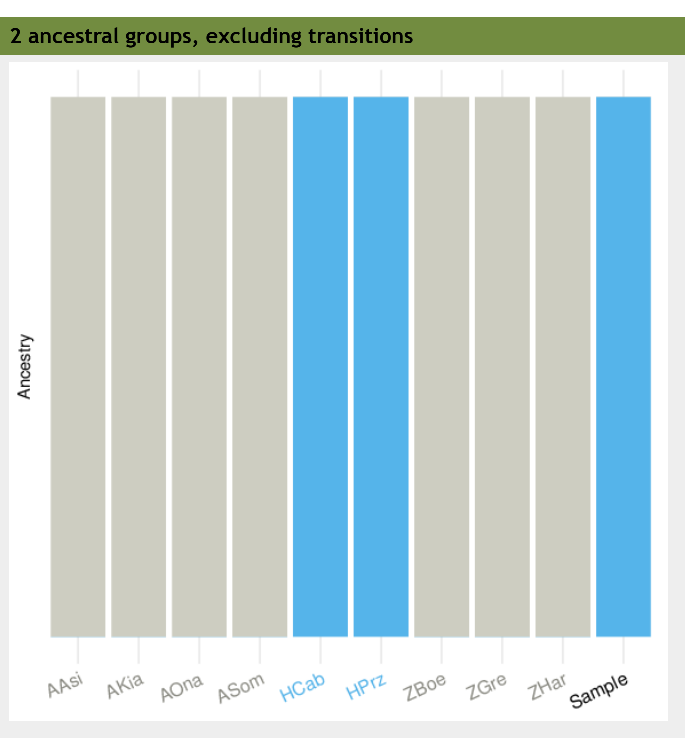

No admixture detected.

3 ancestral groups

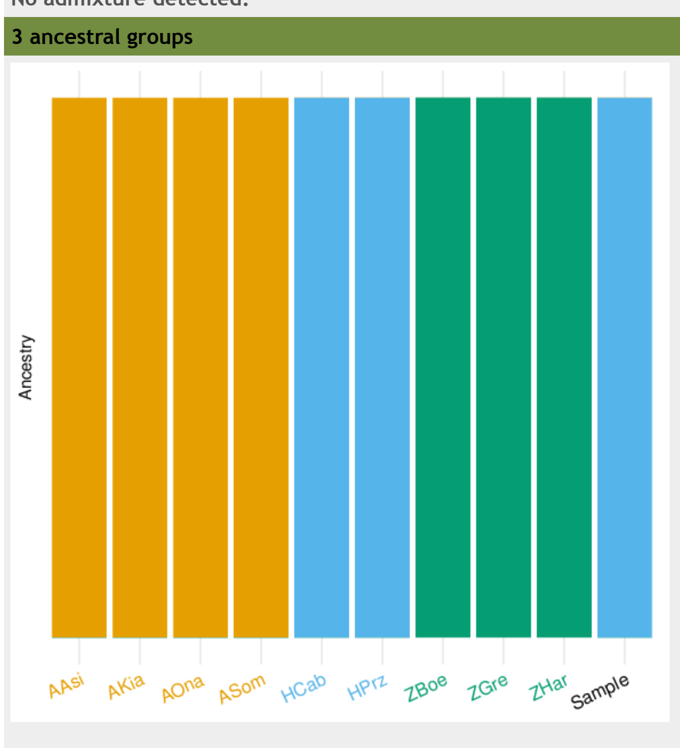

No admixture detected.

3 ancestral groups, excluding transitions

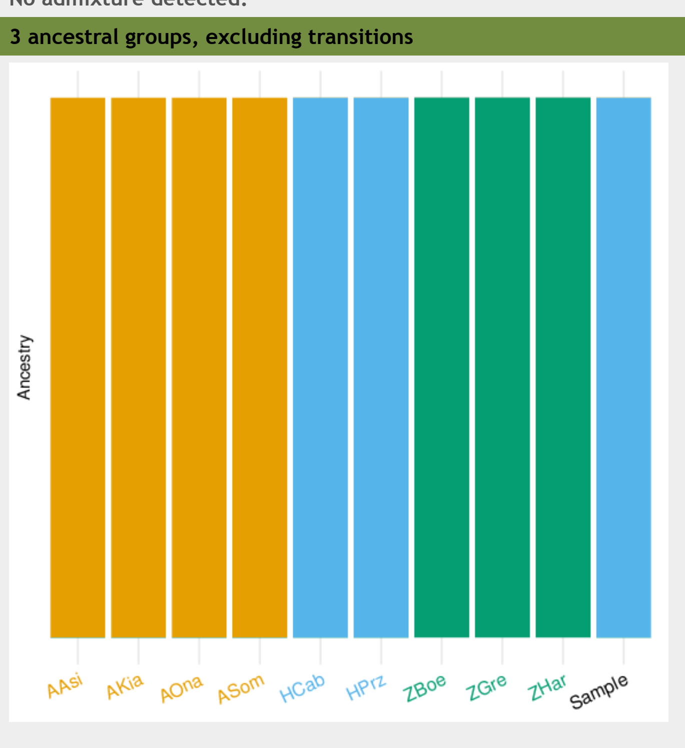

No admixture detected.

PCA PLOTS

Principal Component Analysis carried out using SmartPCA v16000, from the EIGENSOFT toolkit.

Including transitions

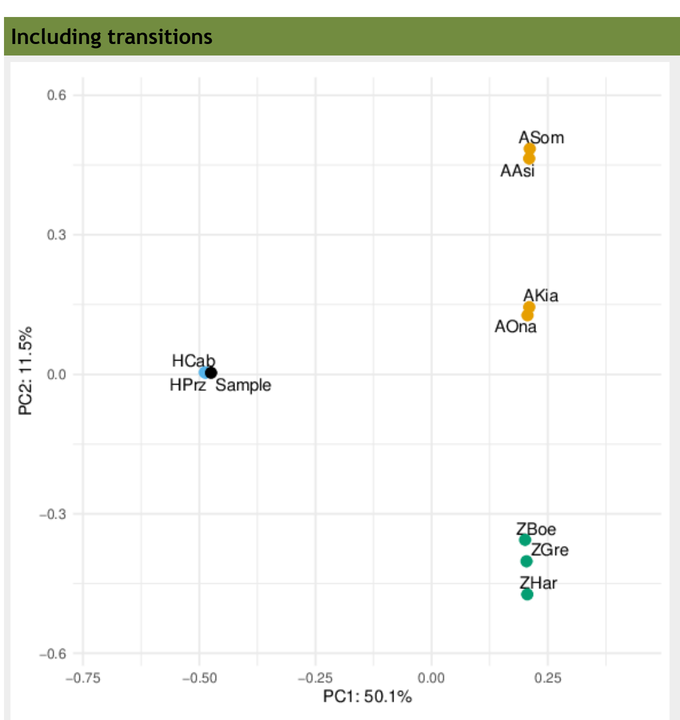

Excluding transitions

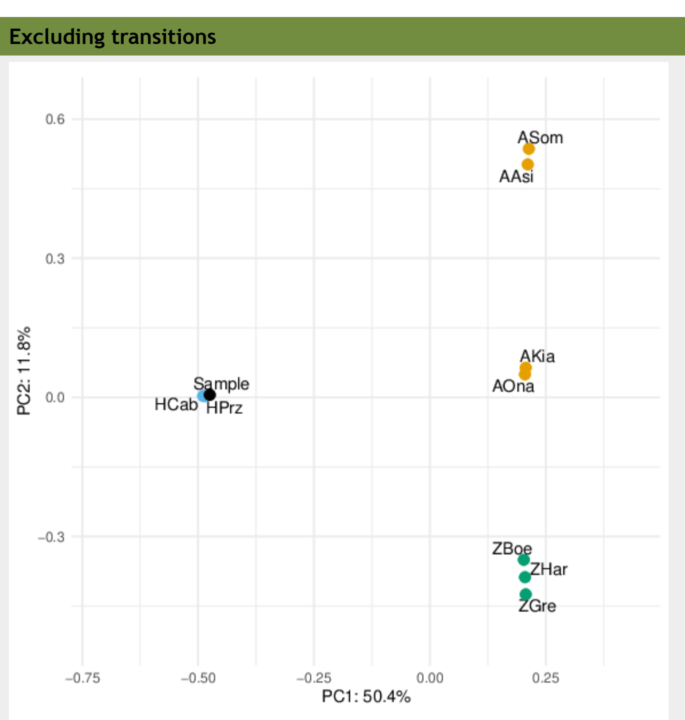

TREEMIX PLOTS

Detection of population mixture using TreeMix v1.13 *[Pickrell and Pritchard 2012]*; parameters were -k 0; -global; and supervised estimation using ancestral groups listed in the Reference Panel.

Including transitions

Edges = 0

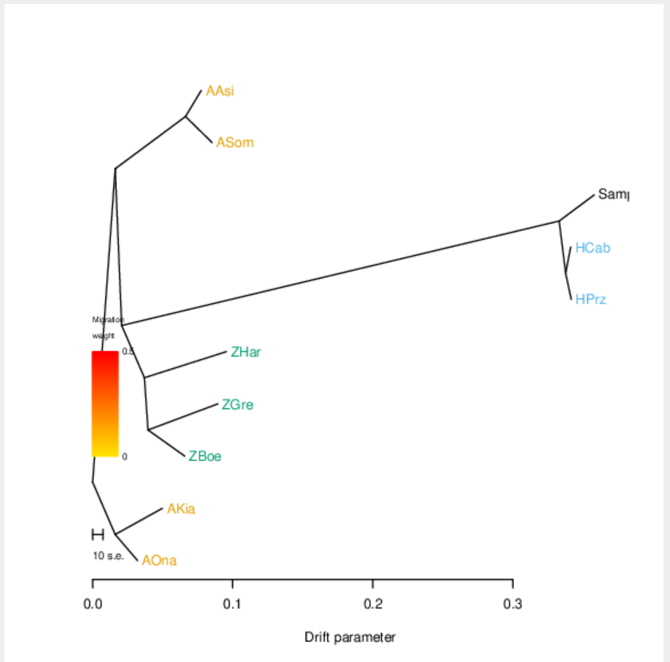

Residuals

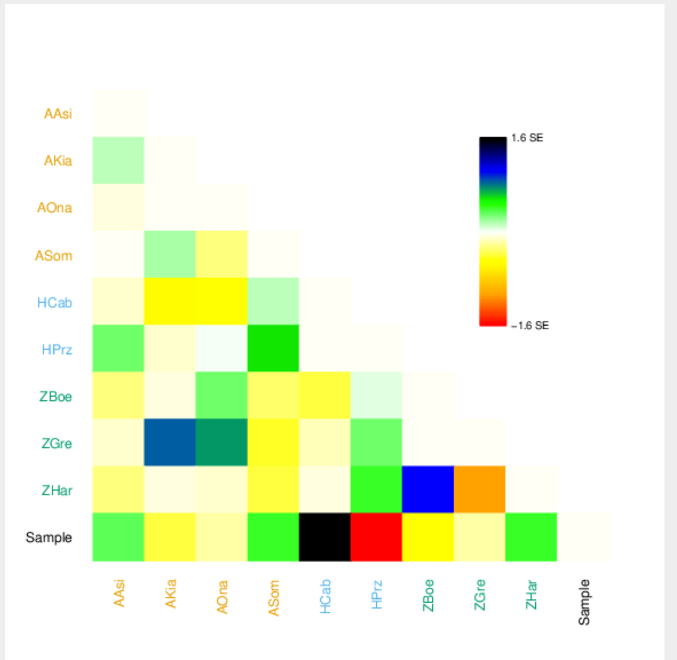

Variance explained by model = 0.999968.

Edges = 1

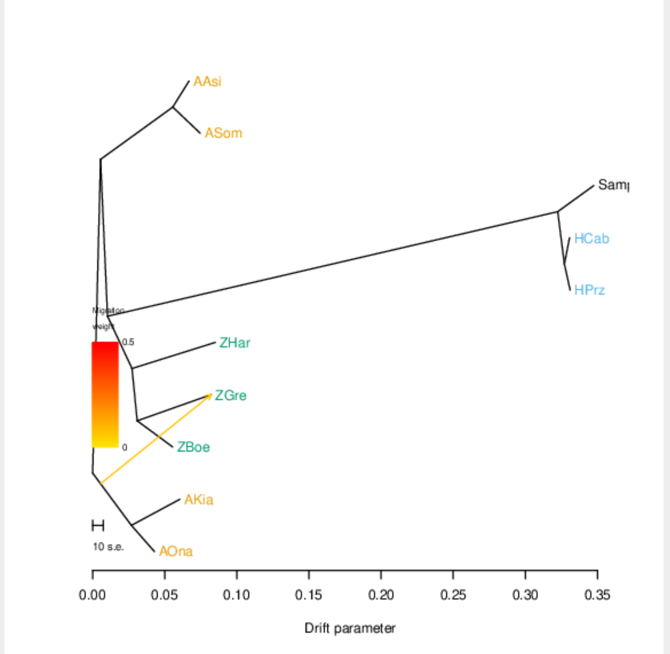

Residuals

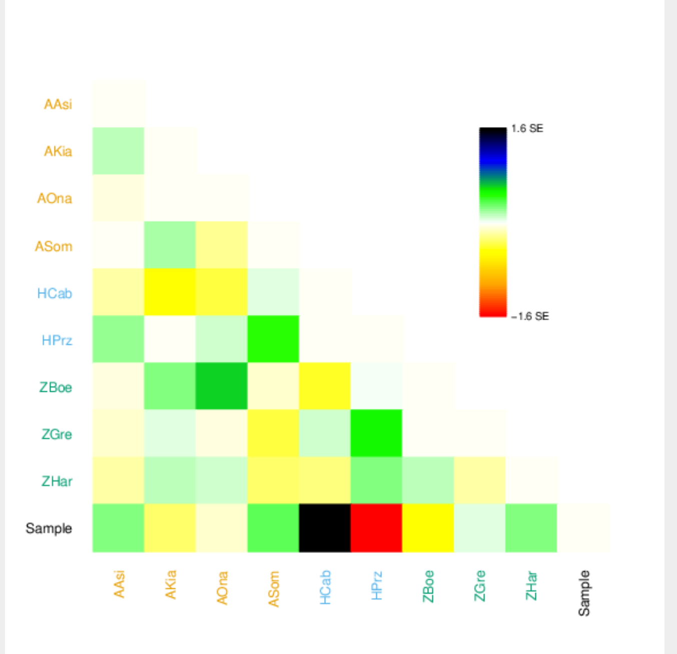

Variance explained by model = 0.999978.

Excluding transitions

Edges = 0

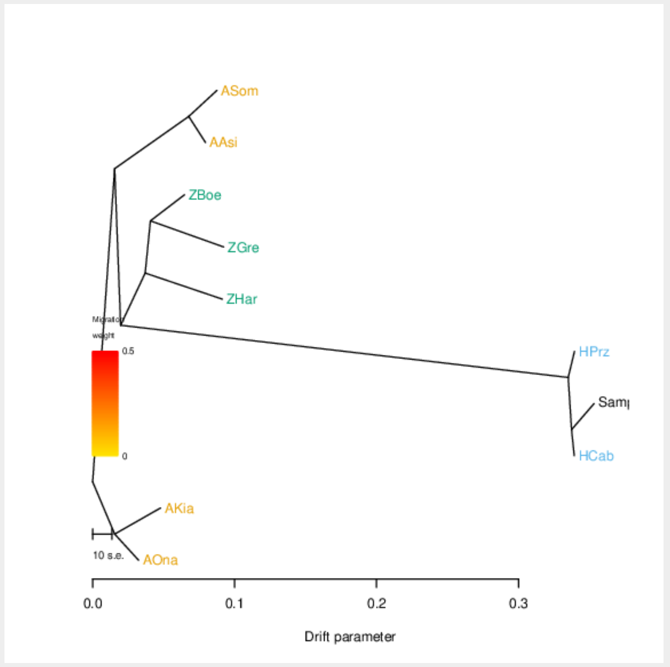

Residuals

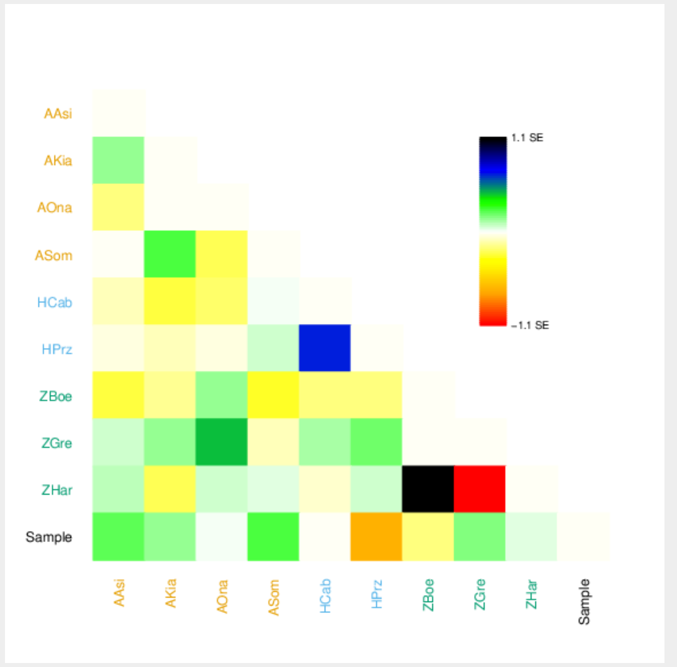

Variance explained by model = 0.999957.

Edges = 1

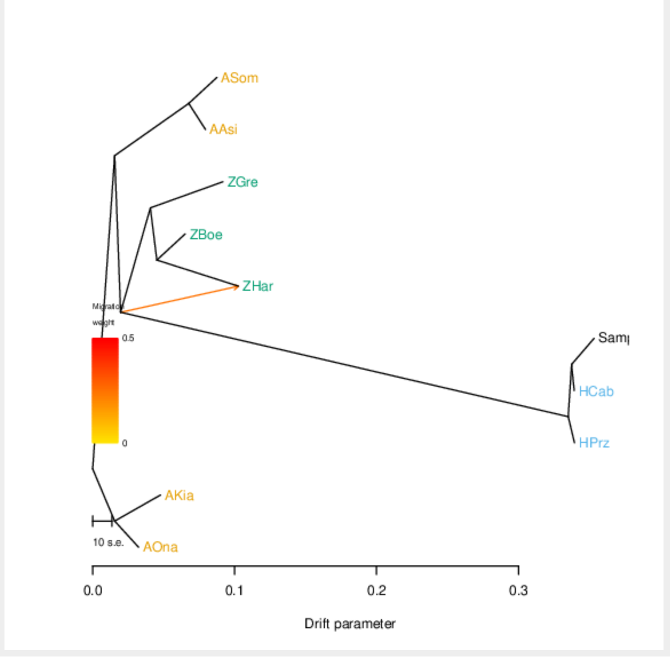

Residuals

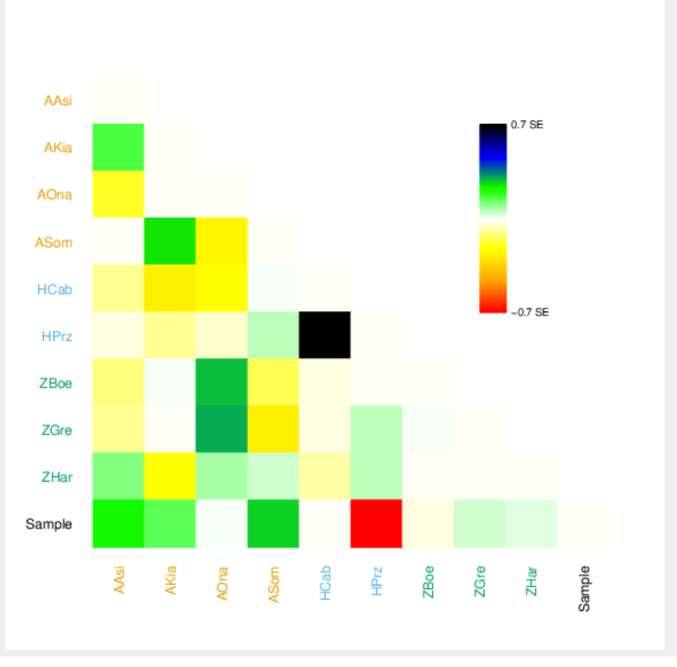

Variance explained by model = 0.999983.

REFERENCES

- Alexander *et al*. "**Fast model-based estimation of ancestry in unrelated individuals**". *Genome Res*. 2009 Sep;19(9):1655-64. doi: 10.1101/gr.094052.109. PMID: 19648217.
- Li *et al*. "**The Sequence Alignment/Map format and SAMtools**. *Bioinformatics*. 2009 Aug 15;25(16):2078-9. doi: 10.1093/bioinformatics/btp352. PMID: 19505943.
- Pickrell and Pritchard. "**Inference of population splits and mixtures from genome-wide allele frequency data**". *PLoS Genet*. 2012;8(11):e1002967. doi: 10.1371/journal.pgen.1002967. PMID: 23166502.
- Purcell *et al*. "**PLINK: a tool set for whole- genome association and population-based linkage analyses**". *Am J Hum Genet*. 2007 Sep;81(3):559-75. PMID: 17701901.
- Stamatakis. "**RAxML-VI-HPC: maximum likelihood-based phylogenetic analyses with thousands of taxa and mixed models**". *Bioinformatics*. 2006 Nov 1;22(21):2688-90. Epub 2006 Aug 23. doi: 10.1093/bioinformatics/btl446. PMID: 16928733.

This report is based on the PLAIN 1.0 design by 6ix Shooter Media, Creative Commons license.
